# The Role of Physical Activity in Harm Reduction among Betel Quid Chewers from a Prospective Cohort of 419,378 Individuals

**DOI:** 10.1371/journal.pone.0152246

**Published:** 2016-04-04

**Authors:** Feng En Lo, Po Jung Lu, Min Kuang Tsai, June Han Lee, Christopher Wen, Chi Pang Wen, Jackson Pui Man Wai, Chwen Keng Tsao, Po Huang Chiang, Shu Yu Lyu, Ko Lu Ma, Ying-Chen Chi, Chu-Shiu Li, Chwen-Chi Liu, Xifeng Wu

**Affiliations:** 1 Department of Leisure and Recreation Management, Asia University, Taichung, Taiwan; 2 Institute of Population Health Sciences, National Health Research Institutes, Miaoli, Taiwan; 3 Department of Radiological Sciences, University of California at Irvine, Irvine, CA, United States of America; 4 China Medical University Hospital, Taichung, Taiwan; 5 Institute of Sport Science, National Taiwan Sport University, Taoyuan, Taiwan; 6 MJ Health Management Institution, Taipei, Taiwan; 7 Department of Public Health, Taipei Medical University, Taipei, Taiwan; 8 Taoyuan Innovation Institute of Technology, Taoyuan, Taiwan; 9 Department of Education & Research, Taipei City Hospital, Taipei, Taiwan; 10 Department of Risk Management and Insurance, National Kaohsiung First University of Science and Technology, Kaohsiung, Taiwan; 11 Department of International Business, Asia University, Taichung, Taiwan; 12 Department of Risk Management and Insurance, Feng Chia University, Taichung, Taiwan; 13 Department of Epidemiology, University of Texas MD Anderson Cancer Center, Houston, TX, United States of America; Tufts University, UNITED STATES

## Abstract

**Objective:**

To assess the benefits of regular exercise in reducing harms associated with betel quid (BQ) chewing.

**Methods:**

The study cohort, 419,378 individuals, participated in a medical screening program between 1994 and 2008, with 38,324 male and 1,495 female chewers, who consumed 5–15 quids of BQ a day. Physical activity of each individual, based on “MET-hour/week”, was classified as “inactive” or “active”, where activity started from a daily 15 minutes/day or more of brisk walking (≥3.75 MET-hour/week). Hazard ratios for mortality and remaining years in life expectancy were calculated.

**Results:**

Nearly one fifth (18.7%) of men, but only 0.7% of women were chewers. Chewers had a 10-fold increase in oral cancer risk; and a 2-3-fold increase in mortality from lung, esophagus and liver cancer, cardiovascular disease, and diabetes, with doubling of all-cause mortality. More than half of chewers were physically inactive (59%). Physical activity was beneficial for chewers, with a reduction of all-cause mortality by 19%. Inactive chewers had their lifespan shortened by 6.3 years, compared to non-chewers, but being active, chewers improved their health by gaining 2.5 years. The improvement, however, fell short of offsetting the harms from chewing.

**Conclusions:**

Chewers had serious health consequences, but being physically active, chewers could mitigate some of these adverse effects, and extend life expectancy by 2.5 years and reduce mortality by one fifth. Encouraging exercise, in addition to quitting chewing, remains the best advice for 1.5 million chewers in Taiwan.

## Introduction

It has been estimated that as many as 600 million people worldwide have the habit of chewing betel quid (BQ). [1] This behavior is most prevalent in South Asia, such as India, Pakistan, Bangladesh, and Indonesia.[1] BQ usually comprises areca nut (areca catechu) and betel leaf (Piper betel) or areca fruit, together with slaked lime, tobacco, or spices inserted.[2] In contrast to the practices of other South Asian countries, betel quid in Taiwan does not contain tobacco; rather, tobacco is consumed separately by smoking cigarettes, resulting in side effects. [2, 3] Chewers in Taiwan were estimated between 1.5 million and 2 million in a population of 8 million adult males. Chewers chewed 5–15 betel quids a day and in addition, more than 90% of chewers also smoked approximately 20 cigarettes. With 5–15 betel quids chewed and 20 cigarettes smoked a day, the chewers suffered from 25–35 times assaults to their oral assaults per day.

The surge in betel quid chewing in Taiwan is a serious problem. Much of the increase has been attributed to the forced opening of the tobacco market by foreign tobacco companies since 1987. [2–5] The forced opening of the cigarette market aided the growth of BQ vendors.[3, 5] Currently, 8 million male chewers in Taiwan affecting the lower socio-economic class, chewed betel quid and widened the existing health disparity. [6, 7]

The harm of chewing betel quid included increase mortality from all cause and from at least 6 types of cancer.[8] It could also come cardiovascular disease, type 2 diabetes mellitus, chronic kidney disease and metabolic syndrome. [9–15] The most frequently encountered pathology was oral cancer and its pre-malignant lesions such as lichenoid changes, leukoplakia, submucous fibrosis. The areca nut and inflorescence piper betle contained carcinogens such as safrole.[2, 16, 17] The addition of lime, a common practice, induces surface injuries in the oral mucosa due to its caustic properties. In addition to oral cancer, chewers are known to have increased cancer at many other sites such as esophagus, liver, pancreas, larynx and lung.[8]

There are four dimensions of physical activity commonly described: Transportation, household chores, physical labor at worksite and leisure-time physical activity (LTPA).[18] Among them, only LTPA is promotable and effort-related and has been most reported with improved health benefits, and extended life expectancy.[18] Its positive effects are extensive with multi-system involvement.[18, 19] The current recommendation for LTPA is at least 30 minutes of daily exercise for 5 days or more per week (150 minutes/week) with moderate intensity.[20, 21] However, our recent study has shown the ability of extending 3 years of life from a daily exercise, not requiring 30 minutes each time but starting from 15 minutes or more of dedicated exercise in moderate intensity.[18]

Preventing chewing and encouraging cessation are the mainstay of strategies for betel quid control. Nevertheless, just like smoking, to quit an addiction like chewing has been met with limited success.[22] The average quit rate was around 6%-12%.[23] Given many chewers who either struggled with cessation or had no interest in quitting, we attempted to assess whether engaging in regular exercise could reduce the chewing harms. A critical question we asked was “to what extent can the harms of chewing BQ be reversed or mitigated by physical activity?”.

A prospective cohort study was conducted to assess the health benefits of engaging in LTPA by chewers who exercised either at a low (15 minutes/day) or recommended volume (30 minutes/day or more). All-cause mortality and life expectancy were assessed as the final outcome, with inactive chewers serving as the comparison group.

## Methods

### Data collection

A cohort of 419,378 individuals (204,533 men and 214,845 women) aged 20 years and older who participated in a standard medical screening program were successively recruited between 1994 and 2008. These individuals were followed-up until the end of 2008, with an average of 8.8 years of observation, by matching their IDs against the National Death File, maintained by the Department of Health in Taiwan.

Each participant completed a self-administered questionnaire which included, among others, assessments of their betel quid chewing and regular exercise habits. Chewers specified their daily amount of BQ chewed from 1 to 5 pieces, 6–9 pieces to ten or more pieces. In this study, current and former chewers were combined as a whole.

In contrast to other Asians using sliced pieces of ripe betel quid, people in Taiwan consume the green unripe areca fruit in its entirety, approximately the size of an olive. Three major types are commonly encountered: Laohwa quid—a split areca nut is sandwiched with the inflorescence (flower) of Piper betle Linn., spiced with red lime; Betel quid—a whole areca fruit is wrapped with betel leaves spread with white lime; Stem quid—a split areca fruit is sandwiched with the stem of Piper betle Linn., spread with white lime. This last type is exclusively consumed by aborigines in a home grown environment.[3]

Three multiple-choice questions were used to ascertain LTPA activities for the past two weeks, including both the duration and intensity of exercise. Exercise intensity was measured by assigning a metabolic equivalent (MET; 1 MET = 1 kcal/hour/kg) value based on Ainsworth’s Compendium.[24] Exercise volume for each individual was derived from the product of intensity and duration, and then placed into one of three categories: inactive (<3.75 MET-h/week), low active (3.75–7.49 MET-h/week or an average of 90 minutes/week), or fully active (≥7.50 MET-h/week or 150 minutes/week or more).[18, 20, 21] In this study, because chewers were less active and the number of chewers were limited, we grouped low active and fully active as one “active” group. The benefits from minimal amount of physical activity by the chewers will be assessed. All participants in this study signed a consent form and institutional review board (IRB) approval was obtained through the "Research Ethics Committee National Health Research Institutes" (approval number: EC0981201-E) in Taiwan. Individual identification was removed and remained anonymous during the entire study process.

### Statistical analysis

Adjusted hazard ratios (HRs) of mortality risk were calculated with the Cox proportional hazards model. Nine variables were adjusted: age, smoking, drinking, physical labor at work, education, BMI, systolic blood pressure, fasting glucose, and total cholesterol. A modified life-table method, relying on age-specific mortality rates, was used to compare life expectancy.[25]

## Results

**Fig 1:**
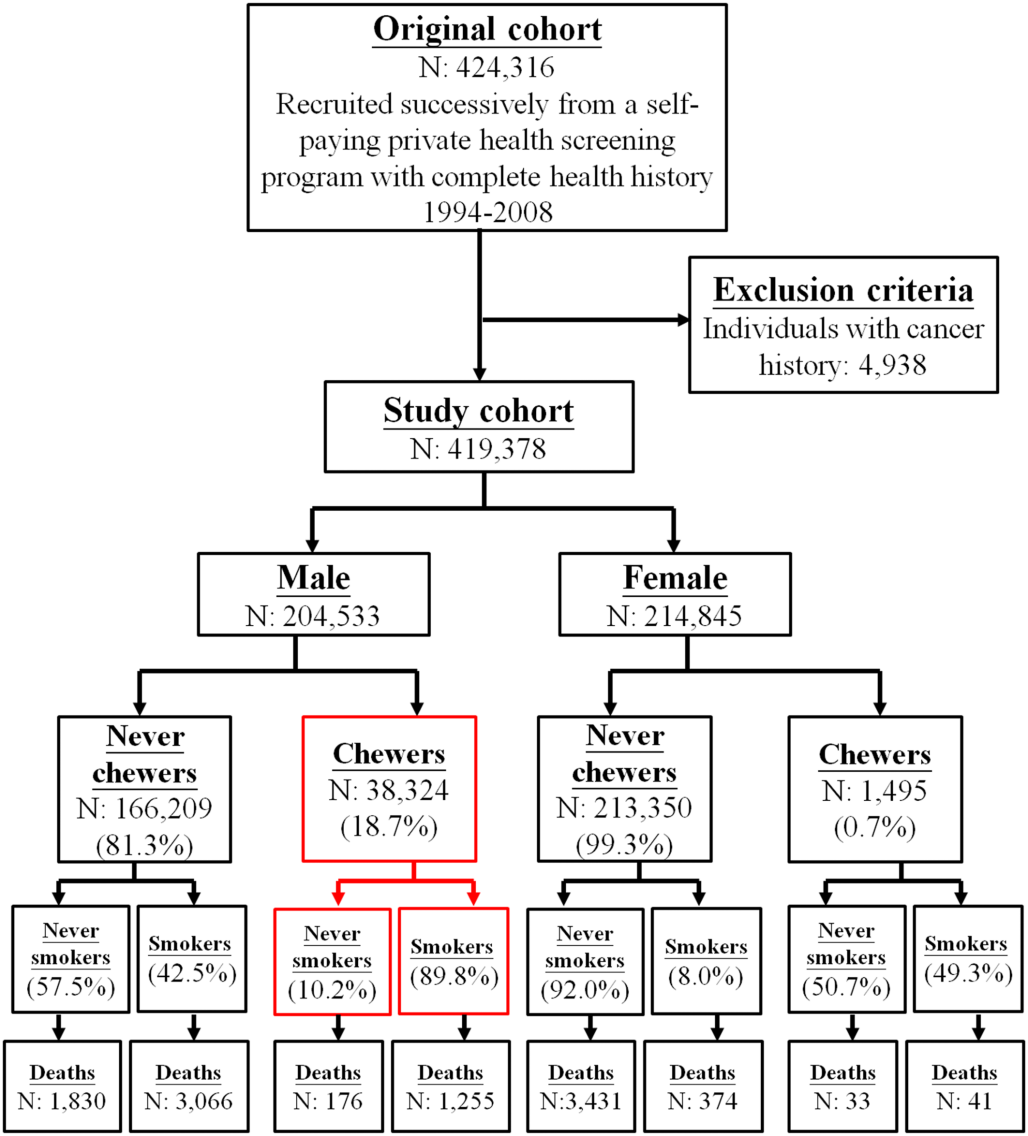
Flow diagram of study subjects by chewing and by smoking status.

Of the 204,533 males in the cohort, 38,324 (18.7%) were chewers (Fig 1). In contrast, very few females were chewers (0.7%). As a result, for this study, we focused on male chewers. More than half of chewers were younger than 40 years old, with few chewers older than age 60 (Table 1). Chewers with smoking made up nearly 90% of the chewers. In contrast, only one third of the cohort smoked. Chewers were less educated and engaged in more physical job at work. Chewers had more drinking habits and were less physically active. They also exhibited higher BMI, higher cholesterol and more diabetes.

**Table 1:** Demographics and clinical characteristics by chewing status.

	Overall	Non-chewers	Chewers
Number of participants ^#^	204533	166209	38324
		(81.3%)	(18.7%)
Age			
20–39	116754	56.8%	58.5%*
40–59	62974	30.0%	34.1%*
≥ 60	24805	13.2%	7.4%
Physical activity status ◊			
Inactive	94750	43.5%	58.7%*
Active (≥ 90 minutes/week)	109783	56.5%	41.3%
Low active (90 minutes/week)	48020	24.4%	19.4%
Fully active (≥ 150 minutes/week)	61763	32.1%	21.9%
Smoking			
Never	97029	57.5%	10.2%
Ex-smoker	21487	10.8%	10.8%
Current smoker	81030	31.8%	79.1%*
Drinking			
Never	129046	71.8%	34.2%
Occasional drinker	46092	20.1%	36.8%
Regular drinker	23890	8.1%	29.0%*
Physical labor at work			
Mostly sedentary	105787	58.1%	32.4%
Sedentary with occasional walking	54662	26.4%	32.3%
Mostly standing or walking	28687	12.1%	24.6%*
Hard labor	9482	3.4%	10.7%*
Educational attainment			
Middle school or below (≤ 9 years)	38153	15.9%	32.1%*
High school (10–12 years)	45161	18.4%	39.6%*
Junior college (13–14 years)	46618	23.9%	19.5%
College or above (≥ 15 years)	72028	41.8%	8.7%
Body mass index			
< 18.5 kg/m^2^	8141	4.0%	4.0%
18.5–24 kg/m^2^	126587	63.0%	57.2%
25–29 kg/m^2^	60798	29.0%	32.9%
≥ 30 kg/m^2^	8930	4.0%	5.9%*
Systolic blood pressure			
< 120 mmHg	90265	43.6%	46.7%
120–139 mmHg	75503	37.2%	35.6%
≥ 140 mmHg or on medication	38717	19.2%	17.7%
Fasting blood glucose			
< 110 mg/dL	178350	87.6%	86.7%
110–125 mg/dL	14141	6.9%	7.0%
≥ 126 mg/dL or on medication	11511	5.5%	6.4%*
Total cholesterol			
< 160 mg/dL	31454	15.2%	16.2%
160–199 mg/dL	85886	42.3%	40.9%
200–239 mg/dL	63349	31.1%	30.5%
≥ 240 mg/dL	23685	11.4%	12.4%*

* Significant characteristics of betel quid chewers by Z-test, *p*<0.05.

^◊^ Physical inactivity was defined “< 3.75 MET-h/week”;

Low activity was defined “3.75–7.49 MET-h/week”;

Fully activity was defined “≥ 7.5 MET-h/week”.

^#^ Only males were shown.

Mortality risks of chewers were compared with non-chewers among the entire cohort and among smokers in Table 2. For all-cause mortality, BQ chewers had nearly doubled the risk among the entire cohort, with HR at 1.92; while one third excess increase among smokers, with HR at 1.35. Chewers also doubled the cancer risks, and when they smoked, the significant increase remained compared to non-chewing smokers. Chewers had 10-fold increase in oral cancer risk compared to nonsmoking and non-chewers after adjusted for age, drinking, physical labor at work, education, BMI, systolic blood pressure, fasting blood glucose, and total cholesterol. Additional increases were found in lung cancer, esophageal cancer, liver cancer and liver diseases. Increases were also found in cardiovascular diseases (CVD), respiratory diseases like COPD, digestive diseases, and diabetes. Smoking chewers compared to smoking non-chewers also had higher risks in most of the diseases mentioned above. Significant mortality risk for injuries were observed compared to non-smoking chewers (HR, 1.92; 95% CI, 1.48–2.49) and smoking chewers (HR, 1.54; 95% CI, 1.17–2.01).

**Table 2:** Mortality risk of chewers in total cohort and among smokers in cohort.

	Total cohort				Smokers in cohort			
	Nonsmoking non-chewers		All chewers		Smoking non-chewers		Smoking Chewers	
Number of participants ^#^	(n = 93248)		(n = 38324)		(n = 69047)		(n = 33470)	
Causes of mortality	Deaths	HRs	Deaths	HRs (95% CI)	Deaths	HRs	Deaths	HRs (95% CI)
**All cause**	1830	1	1431	1.92 (1.77–2.07)*	3066	1	1255	1.35 (1.25–1.46)*
**All cancer**	641	1	588	2.10 (1.86–2.38)*	1242	1	522	1.33 (1.18–1.50)*
Oral cancer	8	1	62	10.72 (5.12–22.4)*	24	1	57	4.01 (2.28–7.05)*
Lung cancer	98	1	116	3.06 (2.26–4.15)*	373	1	104	1.03 (0.80–1.33)
Esophagus cancer	11	1	38	3.00 (1.48–6.04)*	39	1	35	1.85 (1.05–3.26)*
Liver cancer	165	1	187	2.35 (1.85–2.97)*	274	1	160	1.40 (1.11–1.76)*
**Cardiovascular disease**	388	1	239	1.80 (1.50–2.17)*	630	1	210	1.38 (1.14–1.67)*
Ischemic heart disease	105	1	67	1.85 (1.31–2.63)*	203	1	58	1.12 (0.78–1.62)
Stroke	153	1	109	2.09 (1.57–2.77)*	245	1	98	1.60 (1.20–2.13)*
**Respiratory system diseases**	114	1	53	1.78 (1.23–2.57)*	267	1	45	1.12 (0.77–1.24)
COPD ◊	34	1	32	3.84 (2.21–6.69)*	113	1	25	1.60 (0.95–2.69)
**Digestive system diseases**	126	1	130	1.90 (1.44–2.51)*	147	1	111	1.74 (1.30–2.34)*
Liver disease	78	1	81	1.62 (1.44–2.32)*	70	1	70	2.02 (1.36–2.99)*
**Diabetes mellitus**	99	1	67	2.69 (1.90–3.82)*	175	1	60	1.37 (0.96–1.95)
**Injuries** ◊	198	1	210	2.07 (1.66–2.59)*	212	1	186	1.53 (1.22–1.93)*
All accident	152	1	148	1.92 (1.48–2.49)*	161	1	133	1.54 (1.17–2.01)*
MVA	88	1	76	1.49 (1.05–2.73)*	82	1	69	1.55 (1.06–2.26)*
NMVA	64	1	72	2.58 (1.76–3.77)*	79	1	64	1.52 (1.04–2.27)*
Suicide	46	1	62	2.62 (1.68–4.08)*	51	1	53	1.53 (0.97–2.42)

* *p*<0.05; Hazard ratios are adjusted for age, drinking, physical labor at work, education, BMI, systolic blood pressure, fasting blood glucose, and total cholesterol in a multivariate Cox model.

^◊^ COPD = Chronic obstructive pulmonary disease;

MVA = Motor vehicle accident; NMVA = Non-motor vehicle accident;

All accident = MVA + NMVA; Injuries = All accident + Suicide.

^#^ Only males were shown.

Table 3 compared the active chewers with the inactive chewers. We also showed active non-chewers with inactive non-chewers. For all-cause mortality, active chewers reduced by 19% (HR: 0.81) and all-cancer mortality by 22% (HR: 0.78). Other than lung cancer (HR:0.64) and diabetes (HR:0.56), reduction in other causes did not reach statistical significance due to small sample size.

**Table 3:** Benefits or reduced mortality for chewers and for non- chewers from being active.

	Chewers						non-chewers			
Number of participants ^#^	Inactive (n = 22496)		Active (n = 15828)		Active smoking chewers (n = 13682)		Inactive (n = 72254)		Active (n = 93955)	
**Causes of morality**	Deaths	HRs	Deaths	HRs (95% CI)	Deaths	HRs (95% CI)	Deaths	HRs	Deaths	HRs (95% CI)
**All cause**	918	1	513	0.81 (0.72–0.91)*	439	0.82 (0.72–0.92)*	2341	1	2751	0.76 (0.71–0.81)*
**All cancer**	381	1	207	0.78 (0.65–0.94)*	174	0.76 (0.63–0.92)*	858	1	1082	0.80 (0.72–0.88)*
Oral cancer	41	1	21	0.71 (0.41–1.22)	20	0.76 (0.44–1.32)	21	1	13	0.41 (0.18–0.93)*
Lung cancer	81	1	35	0.64 (0.41–0.99)*	29	0.58 (0.37–0.92)*	216	1	265	0.84 (0.69–1.04)
Esophagus cancer	26	1	12	0.85 (0.40–1.79)	10	0.84 (0.39–1.80)	20	1	30	1.06 (0.56–2.00)
Liver cancer	123	1	64	0.76 (0.55–1.05)	48	0.77 (0.49–0.99)*	209	1	247	0.85 (0.69–1.04)
**Cardiovascular disease**	141	1	98	1.07 (0.80–1.42)	85	1.11 (0.82–1.49)	499	1	559	0.66 (0.57–0.76)*
Ischemic heart disease	41	1	26	0.94 (0.54–1.65)	21	0.96 (0.53–1.72)	158	1	160	0.62 (0.48–0.81)*
Stroke	60	1	49	1.31 (0.85–2.00)	44	1.39 (0.90–2.14)	188	1	226	0.73 (0.59–0.92)*
**Respiratory system disease**	33	1	20	0.63 (0.32–1.22)	19	0.72 (0.37–1.39)	186	1	209	0.68 (0.54–0.85)*
COPD ◊	23	1	9	0.40 (0.16–1.03)	8	0.47 (0.18–1.19)	72	1	82	0.69 (0.47–1.00)
**Digestive system disease**	87	1	43	0.82 (0.55–1.21)	36	0.77 (0.51–1.15)	142	1	150	0.68 (0.52–0.88)*
Liver diseases	55	1	26	0.82 (0.50–1.35)	22	0.77 (0.46–1.29)	75	1	88	0.76 (0.53–1.09)
**Diabetes mellitus**	46	1	21	0.56 (0.31–0.99)*	18	0.61 (0.34–1.10)	133	1	158	0.85 (0.65–1.12)
**Injuries** ◊	139	1	71	0.75 (0.56–1.02)	63	0.80 (0.59–1.09)	200	1	227	0.97 (0.78–1.20)
All accident	95	1	53	0.77 (0.54–1.09)	47	0.81 (0.56–1.16)	158	1	169	0.87 (0.68–1.11)
MVA	47	1	29	0.80 (0.49–1.32)	26	0.87 (0.53–1.45)	79	1	100	1.10 (0.79–1.55)
NMVA	48	1	24	0.74 (0.45–1.21)	21	0.75 (0.44–1.26)	79	1	69	0.66 (0.46–0.95)*
Suicide	44	1	18	0.71 (0.40–1.27)	16	0.77 (0.43–1.40)	42	1	58	1.38 (0.88–2.15)

*p<0.05; Hazard ratios are adjusted for age, smoking, drinking, physical labor at work, education, BMI, systolic blood pressure, fasting blood glucose, and total cholesterol in a multivariate Cox model when appropriate.

“Active” was defined “≥ 3.75 MET-h/week”.

^◊^ COPD = Chronic obstructive pulmonary disease;

MVA = Motor vehicle accident; NMVA = Non-motor vehicle accident;

All accident = MVA + NMVA; Injuries = All accident + Suicide.

^#^ Only males were shown.

**Fig 2:**
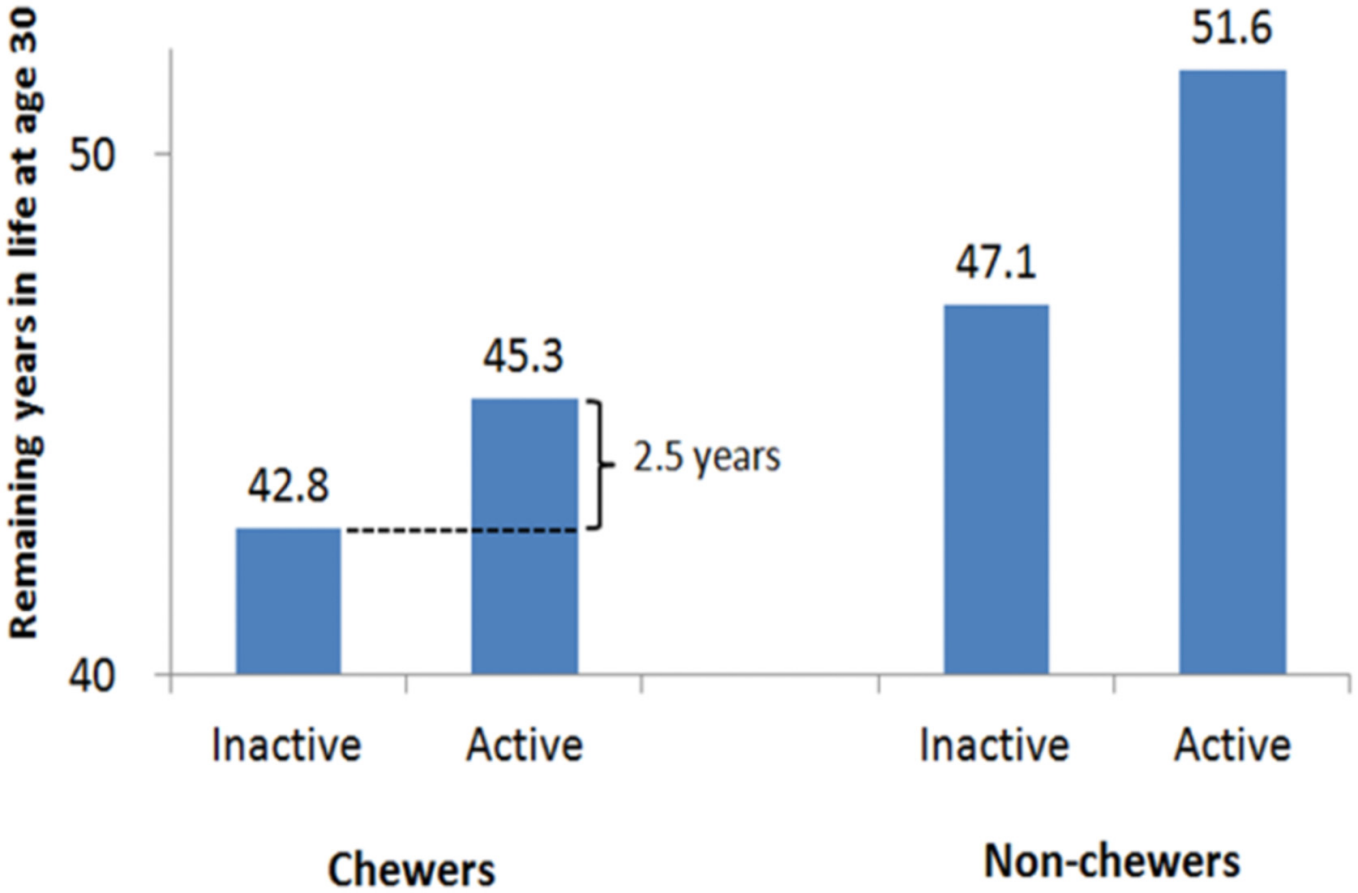
Differences in remaining years in life by chewing status and by physical activity status (males at age 30).

Inactive chewers at age 30 had their lifespan shortened by 4.3 years, when compared to “inactive” non-chewers (Fig 2 and Table 4). Active chewers improved their life span by 2.5 years, from 42.8 years to 45.3 years, but still fell short of “inactive” non-chewers by 1.8 years and “active” non-chewers by 6.3 years.

**Table 4:** Comparison of life expectancy among inactive chewers, active chewers, and general population.

	Inactive chewers ^[A]^		Active chewers ^[B]^		non-chewers ^[C]^					
					non-chewers		Inactive ^[C1]^		Active ^[C2]^	
Age	deaths	life span	deaths	life span	deaths	life span	deaths	life span	deaths	life span
20	0	52.3	0	54.8	12	58.6	4	56.6	4	61.2
25	17	47.4	12	49.9	45	53.9	20	51.9	15	56.5
30	36	42.8	14	45.3	70	49.1	37	47.1	15	51.6
35	43	38.2	22	40.5	104	44.2	60	42.2	24	46.7
40	62	33.4	46	35.7	126	39.3	74	37.4	30	41.9
45	83	28.8	47	31.1	146	34.5	75	32.6	38	37.0
50	80	24.5	32	26.6	188	29.7	99	27.8	32	32.2
55	109	20.2	62	22.0	318	25.1	166	23.3	61	27.5
60	159	16.3	80	18.0	477	20.7	258	19.0	87	23.0
65	153	12.8	81	14.2	710	16.6	354	15.0	120	18.7
70	105	9.5	60	10.6	981	12.7	440	11.2	182	14.8
75	42	6.5	31	7.3	924	9.4	370	7.9	168	11.3
80	20	3.6	18	4.0	637	6.3	245	4.9	90	8.1
85	9	0.9	8	1.3	354	3.5	139	2.2	62	5.4

At age 30 males:

Difference in life span: [B]–[A] = 2.5

95% confidence interval: [A] = 42.1 to 43.5, [B] = 43.7 to 46.9, [C] = 49.0 to 49.2, [C1] = 46.9 to 47.3, [C2] = 51.4 to 51.7

## Discussion

In this large prospective cohort, we showed that BQ chewers had an increased risk of mortality from almost every disease across every system of the body. Chewers doubled the all-cause mortality and had a ten-fold increase in oral cancer risk. They also increased cardiovascular disease and diabetes mortality. The finding that the risk of mortality for chewers increases for CVD and diabetes is consistent with previous studies.[9, 11, 26, 27] One out of two chewers died from chewing-related diseases, with HR for all-cause at 1.92. With nearly 1.5 million people involved in this behavior, the disease burden on society in Taiwan from mortality and morbidity is devastating when considering the financial costs and productivity loss involved.[4]

In this study, we reported the health effect of regular exercise on BQ chewers based on mortality differentials. We found that those who self-reported exercise extended their life span by 2.5 years. This is to say, by engaging in at least 15 minutes of exercise every day, chewers could mitigate considerable amount of harms caused by chewing, 2.5 years out of 4.3 years for the inactive chewer, and to reduce mortality by one fifth (19%). This finding is encouraging news for the struggling chewers who had difficulty in quitting. It should be noted that BQ chewing per se had serious health consequences, including a 2-fold increase in mortality risk and shortened life by as many as 8.8 years (Fig 2). From a harm reduction perspective, it is understandable that daily exercise could only reverse part of the harm from someone chewing 5–15 times a day. These findings suggest that cessation of chewing must remain a top priority, while encouraging chewers to engage in regular physical activity as an important remedial process.

The majority of chewers were inactive, with 78% not meeting the recommended LTPA. Chewers thus have a large opportunity to engage in exercise to take advantage of its benefits.[28, 29]With regular exercise at 15 minute/day or more, life span of chewers could be extended due to the combined effect of improved physical and mental well-being. However, exercise benefits were limited when compared with the harm of chewing. Of course, quitting chewing or early screening and timely intervention for early BQ-related cancer could also extend their lives.

By increasing the BQ price, warning chewers of its harms, and banning its marketing promotion, a series of actions proposed by “MPOWER” from the World Health Organization’s (WHO’s) in global tobacco control,[30] the growing tide in BQ chewing could be curbed. However, due to limited global experience or success stories in betel quid control and domestic political pressure (i.e., to avoid offending the voters who consume the substance), the government has not been aggressive in its effort to curb BQ consumption. Promoting exercise can be an effective way to combat this serious health menace, a move far better than inaction so far for decades.

There are important limitations to this study. First, this is a prospective cohort study and not a clinical trial; thus, causal relationships should not be over-interpreted. Chewers tended to be less active than non-chewers, and encouraging chewers to exercise can only be beneficial and cause no harm. The reasons why chewers were more inactive are not clear but could be speculated. With smoking 15 cigarettes and chewing 15 times of BQ a day, chewers had less time or efforts to exercise. They were also less educated with fewer friends who could exercise with them. However, exercise is not a panacea, and cessation remains the first intervention to pursue. Second, in this study, only leisure-time physical activity was considered, which represented only one aspect of physical activity. However, in our analysis, we have controlled the physical labor at work. Furthermore, the mental and physiological benefits from LTPA have been reported to be larger than the other three domains.[31, 32] Third, we used chewing history data gathered from the initial examination and did not follow-up to monitor any possible changes in this behavior. However, as most of the individuals in the study were past the age of initiation for chewing, with less than 13% started after age 30, few non-chewers at the commencement of the study picked up chewing during the study period. [3] Active chewers could quit chewing similar to or quit even more than inactive chewers, as found in active smokers who quit more when compared to inactive smokers.[33] Nevertheless, the benefits of exercise were large and significant, regardless of its mechanism. Fourth, the level of exercise was self-reported and could not be verified. Because people tend to overstate their exercise habits,[34] our results could be an underestimate of its beneficial effects. Fifth, the follow-up of this cohort for vital status relied solely on matching with National Death File. This assumed that all deaths of the cohort were captured in the National Death File, and no errors of recordings of individual identifications were made. In reality, some errors must have occurred and some deaths were not reported to the National Death File, although it was estimated to be minimal.[35] As a result, we must have under-estimated the number of deaths in this cohort. However, the under-reporting of deaths probably occurred at a similar rate for the chewers as for the never chewers, and for the “active” as for the “inactive”, and the final hazard ratios or differences in life expectancy would have remained the same as we reported.
